# Enhanced ethanol production via electrostatically accelerated fermentation of glucose using *Saccharomyces cerevisiae*

**DOI:** 10.1038/srep15713

**Published:** 2015-10-30

**Authors:** Anup Sam Mathew, Jiapeng Wang, Jieling Luo, Siu-Tung Yau

**Affiliations:** 1 Department of Electrical and Computer Engineering, Cleveland State University, Cleveland, Ohio 44115, USA; 2 The Applied Bioengineering Program, Cleveland State University, Cleveland, Ohio 44115, USA

## Abstract

The global demand for ethanol as an alternative fuel continues to rise. Advancement in all aspects of ethanol production is deemed beneficial to the ethanol industry. Traditional fermentation requires 50–70 hours to produce the maximum ethanol concentration of 7–8% (v/v). Here we demonstrate an electrostatic fermentation method that is capable of accelerating the fermentation of glucose using generic *Saccharomyces cerevisiae* as the fermenting microorganism to produce ethanol. The method, when applied to the batch fermentation of 1 liter fermenting mixture containing dry yeast without pre-culture, is able to achieve ethanol yield on the high gravity level (12.3% v/v) in 24 hours. The fermentation results in almost complete consumption of glucose. With pre-cultured yeast, ethanol yield can reach 14% v/v in 20 hours. The scale-up capability of the method is demonstrated with 2 liter fermenting mixture. The method does not consume external energy due to its electrostatic nature. Our results indicate the applicability of the fermentation technique to industry applications.

Ethanol as a renewable and environment-friendly fuel has attracted significant interests^1^. In recent years, ethanol production in the US has augmented from 4.89 billion gallons in 2006 to 14 billion gallons in 2011, indicating an almost tripled increase^2^. Similar increases have occurred in other countries such as China and India. The dramatic increase is mainly due to the fast depletion of global oil resource and worsening environmental conditions. Traditionally, industrial production of ethanol for fuel use is achieved by the fermentation of sugar using cultured generic *Saccharomyces cerevisiae (S. cerevisiae)*, the so-called baker’s yeast, as the fermenting microorganism^3^. As described in a comprehensive review^4^, traditional fermentation usually takes place at 32–34 °C with an initial glucose concentration of less than 180 g/l and yields a maximum ethanol concentration of 7–8% (v/v), which is imposed mainly by the ethanol stress of yeast. To achieve higher ethanol yield, high gravity (HG) ethanol fermentation with initial glucose at the 180–220 g/l level has been demonstrated to achieve a maximum ethanol concentration of 10–12% (v/v). The time required to reach the maximum ethanol concentration for the traditional and HG fermentation methods is usually 50–70 hours^4^,^5^. HG ethanol fermentation can be performed using only specific strains of *S. cerevisiae* or different microorganisms as the fermenting microorganism. These particular strains or microorganisms have higher thresholds for ethanol stress and therefore can tolerate higher ethanol concentrations. It has been shown that these specific fermenting microorganisms can even yield ethanol concentrations at the 15% (v/v) level with initial glucose of more than 250 g/l. This very high gravity (VHG) fermentation has been demonstrated with *Zymomonas mobilis* (*Z. mobilis*)^6^ and the Z5 strain of *S. cerevisiae*^7^.

To keep pace with the increasing demand for ethanol, the ethanol industry is expected to provide improved productivity. Recently, an electrochemical-electrostatic technique has been developed to control the kinetics of glucose metabolism in *S. cerevisiae*^8^. The technique was used to control short-term (3 hours) production of ethanol on the microliter level. In the present work, a pure electrostatic version of this technique, wherein a voltage is applied to the fermentation mixture without causing a current, is used to accelerate 1–2 liter batch fermentation of glucose with generic *S. cerevisiae.* Without pre-culture of the yeast, the technique produced ethanol beyond the HG level in 24 hours. With pre-cultured yeast, the ethanol yield approached the VHG level in 20 hours. The electrostatic technique does not consume external energy, since no electric currents are involved.

## Results

**Figure 1:**
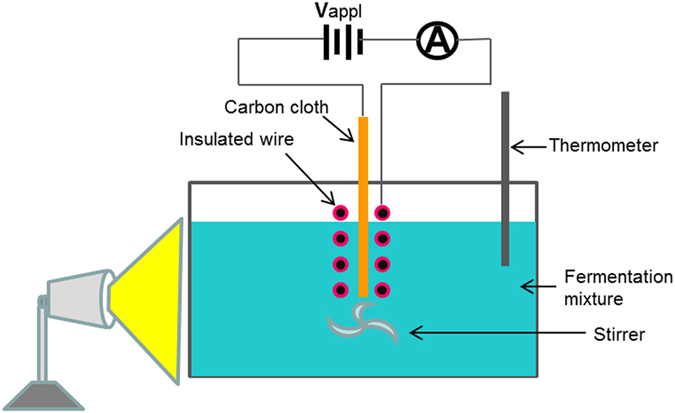
Experimental system of the electrostatic fermentation. The black circles are the metallic wire, which is covered by the red insulator.

**Figure 2:**
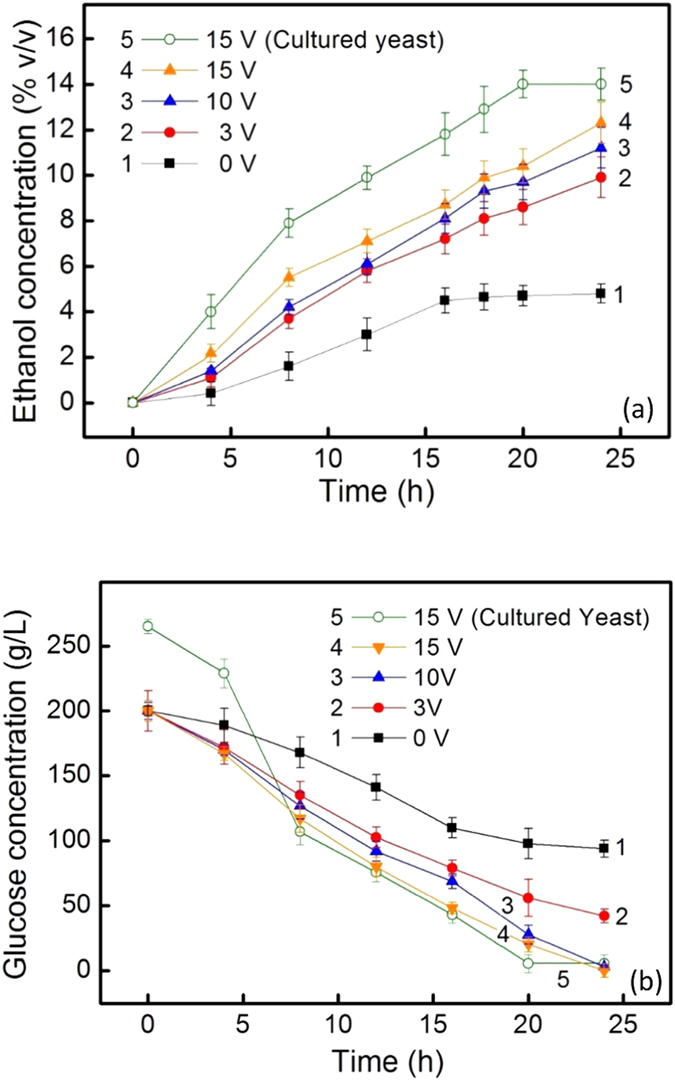
Simultaneous measurements of ethanol and glucose concentrations. (**a**) Production of ethanol starting with dry *S. cerevisiae* is monitored for different V_appl_ values. Curve 5 is obtained with inoculated *S. cerevisiae*. (**b**) Simultaneous measurements of glucose concentration for the curves in (**a**).

The experimental system is described in Fig. 1. The electrostatic fermentation was conducted at 30 °C with batch 1-liter mixtures, containing 200 g/l glucose, which is within the HG range, 12 g/l of dry *S. cerevisiae* (without pre-culture) and water. The area of the carbon cloth electrode was 308 mm^2^. Throughout the fermentation, the electrode circuit was carefully monitored using an ammeter to ensure the absence of current. Curves 1–4 in Fig. 2(a) show the ethanol yields measured during a 24-hour fermentation period for different V_appl_ values. Curves 1–4 in Fig. 2(b) show the simultaneous changes in glucose concentration in the fermentation mixture. Curve 1 in Fig. 2(a) shows that the final ethanol yield of the control fermentation obtained without applying V_appl_ is 4.8% (v/v) with a slow increasing trend. Curve 1 in Fig. 2(b) shows that the corresponding glucose concentration is 95 g/l with a deceasing trend. With increasing V_appl_, the fermentation proceeds at progressively faster rates as indicated by Curves 2–4 in Fig. 2(a). Curve 4 shows that, with V_appl_ = 15 V, after 24 hours of fermentation, the ethanol yield is 12.3%, which is slightly above the HG level. The corresponding glucose concentration as shown in Fig. 2(b) is 0.5 g/l.

**Figure 3:**
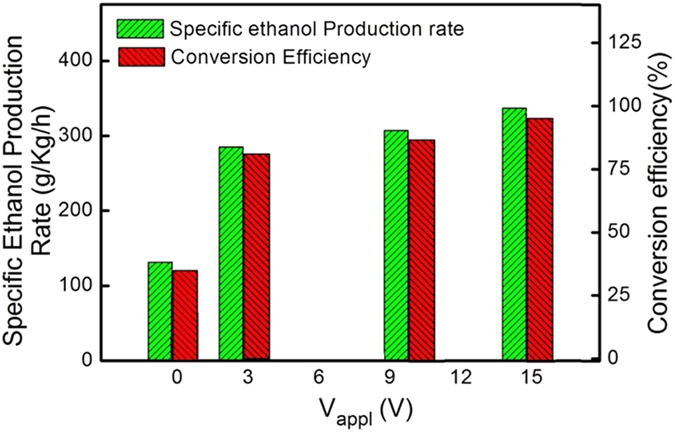
SEP rate and GEC efficiency of the electrostatic fermentation are dependent on V_appl_.

The V_appl_-dependent fermentation can be better quantified by plotting the specific ethanol production (SEP) rate and the glucose-ethanol conversion (GEC) efficiency as shown in Fig. 3. The SPE rate defined as the final ethanol weight (grams) per weight of dry yeast (kilogram) per fermentation time (hour) indicates the productivity of a fermentation process. The maximum (100%) GEC efficiency is obtained according to the theoretical 1-to-2 mole relation in the reaction: C_6_H_12_O_6_→2C_2_H_5_OH + 2CO_2_. As V_appl_ is increased from 0 V to 15 V, both the SEP rate and the GEC efficiency are enhanced. Applying V_G_ = 15 V makes the SEP rate experience a 2.6-fold increase from the control. The enhanced SEP rate implies that V_appl_ makes each *S. cerevisiae* cell produce ethanol more efficiently. This observation is echoed by the improved GEC efficiency. In general, fermentation of glucose using *S. cerevisiae* may produce, in addition to ethanol, acetic acid and butyric acid as end products, implying the adoption of other pathways^9^. These deviations from the ethanol pathway result in less GEC efficiency. Figure 3 shows that the application of V_appl_ increases the GEC efficiency toward 100%. For example, in the case of V_appl_ = 15V, the GEC efficiency reaches 95% and glucose, as shown in Fig. 2(b), becomes almost completely consumed with a final concentration of 0.5 g/l, which is significantly less than the desired 2–5 g/l level for practical ethanol fermentation^4^. This effect is possibly caused by a switch to a more favorable pathway for ethanol production.

**Figure 4:**
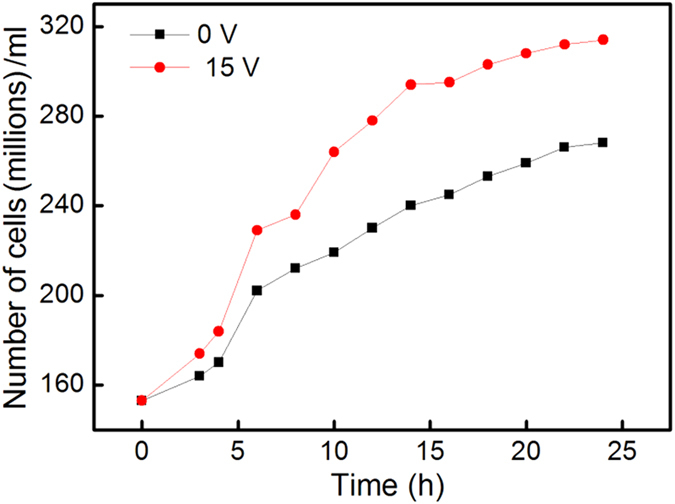
Effect of V_appl_ on the growth of *S. cerevisiae.*

To gain insight into the significant increase in the ethanol yield caused by V_appl_, the growth of the yeast was characterized using optical density measurements performed with a spectrophotometer. In the present work, the fermentation was started with dry *S. cerevisiae* without pre-culture. The lag phase of yeast budding, wherein dry yeast undergoes preparation for reproduction and its normal functioning is not fully restored, can be as long as several hours^10^. Figure 4 shows the yeast concentration profiles of two growth processes obtained with V_appl_ = 0 and 15 V, respectively. In order to correlate the results of the growth processes to Curve 1 and Curve 4 of Fig. 2(a), the same conditions were used for these processes as in the fermentation, namely, 1-liter mixtures, containing 200 g/l glucose, 12 g/l of dry *S. cerevisiae* and water at 30 °C. The two profiles show an initial 4-hour lag phase characterized by a slow growth followed by a sudden 14–21% increase in growth. Comparing the two profiles after the lag phase indicates that the effect of V_appl_ = 15 V is to cause a 1.1 times average increase in cell concentration above that of the V_appl_ = 0 V growth. Therefore, the observed average 2.6-fold increase in ethanol yield indicated by Curve 4 above Curve 1 in Fig. 2(a) is caused, to a less degree, by the increased cell growth. In other words, other mechanisms are also responsible for the enhanced ethanol yield.

Another interesting observation is the fermentation in the lag phase of *S. cerevisiae*. In Fig. 2(a) Curves 1–4 are characterized by a substantial increase in ethanol yield rate after the 4^th^ hour, indicating the end of the 4-hour lag phase. Curve1 in Fig. 2(a) obtained without V_appl_ shows that, at the end of the lag phase, the ethanol yield is 0.4% while the corresponding ethanol yields in Curves 2–4 obtained with V_appl_ approaches 2%. This observation indicates that V_appl_ enhances ethanol production even in the lag phase. This conclusion is confirmed by examining Curves 1–4 in Fig. 2(b), which indicate that V_appl_ enhances the simultaneous glucose consumption during the lag phase.

**Figure 5:**
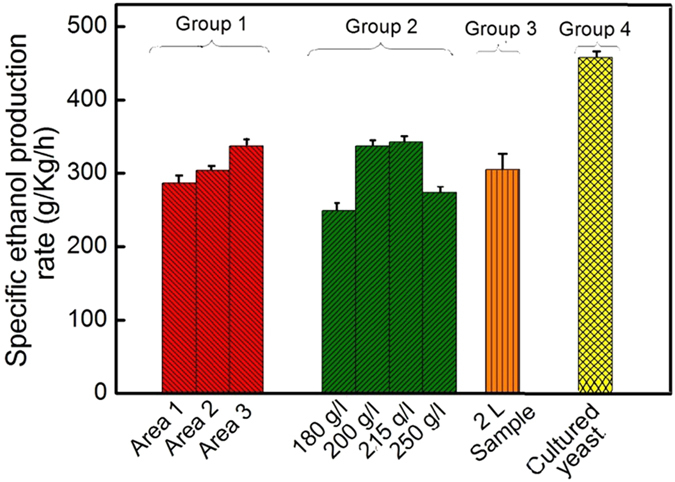
SEP rate of electrostatic fermentation under different conditions. Group 1: Ethanol production is increased as the electrode area is increased from 146 cm^2^ (Area 1) through 228 cm^2^ (Area 2) to 308 cm^2^ (Area 3). Group 2: The effect of initial glucose concentration on ethanol production. Group 3: Similar ethanol production to is obtained using 2-liter fermentation mixture. Group 4: The fermentation performed using cultured *S. cerevisiae* approaches the ethanol yield on the VHG level.

Several other types of measurements have been made to show the capability of the fermentation technique and the feasibility for its further improvement. Figure 5 shows four groups of SEP rates obtained under different conditions, including variations in electrode area, initial glucose concentration, fermentation volume and pre-culture of yeast. These measurements, except group 4, were made with 12 g/l of dry *S. cerevisiae* and V_G_ = 15 V at 30 °C for 24-hour fermentation. Group 1, obtained with 200 g/l glucose, shows that the ethanol yield depends on electrode area. The ethanol productivity increases with increasing electrode area. The three columns from left to right were made with electrodes with areas of 146 cm^2^, 228 cm^2^ and 308 cm^2^, respectively. The third column in this group is the same as the second column in Group 2 obtained using Curve 4 in Fig. 2(a). The results of Group 1 imply that the time needed to produce the present maximum ethanol yield can be further shortened or the productivity can be further enhanced with the electrode area increased beyond the present size of 308 cm^2^. The dependence of ethanol yield on electrode area shows the necessity of V_appl_. Group 2 shows the dependence of ethanol yield on initial glucose concentration within the HG range. It shows that by increasing the initial glucose from 180 g/l to 215 g/l, the SEP rate is increased with the final ethanol yield at 215 g/l being 12.6% (v/v). However, at 250 g/l, a concentration beyond HG, the rate falls back with a yield of 10% (v/v) due to ethanol stress. Group 3 shows the SEP rate of the fermentation of a 2-liter fermentation mixture performed with a 440 cm^2^ electrode under otherwise identical conditions as those in Group 1. The fermentation has an ethanol yield of 11% (v/v) and the SEP rate is almost the same as that of the second column in Group 1 obtained with 228 mm^2^. Therefore, the results suggest a scalable process.

Group 4 shows the SEP rate for pre-cultured *S. cerevisiae* calculated using Curve 5 in Fig. 2(a). Curve 5 was obtained with inoculated *S. cerevisiae* cells. The inoculation was performed by adding 12 g of dry *S. cerevisiae* to a culture mixture that contained 20 g of peptone and 20 g of glucose and 400 ml of water. The mixture was kept at 30 °C for 8 hours. Then, fermentation was performed by adding a solution containing 265 g of glucose and 600 ml of water to the culture mixture and applying V_appl_ = 15 V. Compared to Curve 4, Curve 5 shows significant further improvement in ethanol yield which reaches 14% (v/v), approaching the VHG range, in 20 hours. The glucose concentration at the 20^th^ hour is 5.1 g/l, indicating a 98% consumption. The result suggests that V_G_ is capable of making significant improvement of the productivity in industrial fermentation, which uses pre-cultured yeast. Compared to the fourth column (250 g/l glucose) in Group 2, the result of Group 4 indicates that the pre-cultured yeast cells are fully developed cells and they may have higher resistance to ethanol stress.

## Discussion

It is mentioned above that mechanisms other than increased cell growth are the main cause of the enhanced ethanol yield. Previously it was demonstrated that a voltage can be used to control the kinetics of glucose metabolism in *S. cerevisiae* under aerobic and anaerobic conditions^8^. The different metabolic pathways in yeast cells all involve redox reactions catalyzed by redox enzymes. For example, the redox reactions of the cellular charges NAD^ + ^and NADH are catalyzed by different dehydrogenases in glycolysis, the Krebs cycle, and the electron transport chain^11^. It was suggested that the voltage polarizes ionic charges in yeast cells to induce electric fields, which lower the tunnel barrier experienced by the transferring electrons associated with the cellular charges. This effect was studied in detail and confirmed with a glucose-glucose oxidase enzymatic biocatalytic system in terms of enhanced electron transfer^12^. This effect resulted in enhanced metabolic rates in the glucose metabolism study^8^, leading to voltage-controlled glucose consumption and correlated production of APT. Similar effects may occur in the present system. If this effect indeed occurs, it will also speed up cellular electron transfer even in the lag phase to rapidly energize the cell. The results will be the simultaneous enhanced cell growth and fermentation.

Figure 2 shows that, with the help of V_appl_, *S. Cerevisiae* is able to achieve ethanol yield on the HG level within 24 hours. The results indicate an apparent higher threshold for ethanol stress. A possible reason is that the ethanol production occurred so rapidly that the yeast was not fast enough in adjusting its metabolism to the toxic environment to exhibit the ethanol inhibition effect. For example, the attack of membranes of organelles and cells by ethanol is a major cause of ethanol inhibition^13^ and this process takes a certain amount of time.

The ethanol yield of *Z. mobilis*, reportedly as high as 97% of the theoretical glucose-to-ethanol conversion, is higher than that of generic *S. cerevisiae* because glucose metabolism in *Z. mobilis* adopts its special Entner–Doudoroff pathway, resulting in a higher metabolic rate^14^. However, because *Z. mobilis* ferment effectively only glucose and its biomass is not suitable to be used as animal feed, this microorganism cannot readily replace *S. cerevisiae* in industrial ethanol production^4^. In this context, the featured electrostatic fermentation technique, when applied to *S. cerevisiae*, appears to provide the fermentation performance of *Z. mobilis* by delivering ethanol production at the HG-to-VHG level. Furthermore, the technique is capable of significantly shortening the fermentation time.

Previously, external voltages have been applied to fermentation via electrochemical bioreactors^15^. A voltage source is connected across the reactor to induce a current in the reactor solution that contains *S. cerevisiae*. In this way, electrons are supplied to the microorganism from the cathode to bring external energy to the microorganism so that it can grow faster to carry out fermentation. The work shows that applying −1.5 V to the fermentation of 120 g/l glucose using *S. cerevisiae* that has been cultured for 16 hours achieves an ethanol yield of 53 g/l (6.72% v/v) in 50 hours. However, the improvement over the control fermentation is only 6 g/l (0.76% v/v). The novelty of the electrostatic method demonstrated here lies in the fact that the electrostatic method does not cause currents so that no energy is consumed during the fermentation process. While conserving energy, the electrostatic method nevertheless achieves high ethanol yield (up to 14% v/v) in a short period of time (20 hours).

This article shows that applying a voltage to a fermentation mixture containing glucose and generic dry *S. cerevisiae* results in the completion of the production of ethanol on the HG level in 24 hours. The electrostatic method does not cause currents so that no energy is consumed during the fermentation process. The accelerated fermentation is confirmed by measuring the simultaneous glucose consumption. The final ethanol yield can reach 12.3%v/v. With pre-cultured *S. cerevisiae*, the electrostatic method is able to complete fermentation at the VHG level in 20 hours with an ethanol yield of 14% v/v. Monitoring the growth of the *S. cerevisiae* cells shows that while the applied voltage increases the growth of the cells, the main cause of the enhanced ethanol production remains open. It is speculated that the applied voltage may induce electric filed in the cell and therefore speeds up the cellular electron transport to enhance the fermentation rate. Studies on the effects of electrode area, initial glucose level and the volume of the fermentation mixture indicate that the productivity of ethanol can be further improved for potential use in ethanol industry. Future studies will be conducted with well-defined genotypes of yeast order to elucidate the exact molecular mechanism. Also, the composition of the fermentation solution will be analyzed to study the dependence of the fermentation pathway on V_appl_.

## Methods

### System and operation

The experimental system is described in Fig. 1. The fermentation cell consists of an electrode assembly and a fermentation mixture contained in a sealed glass container. The electrode assembly consists of a piece of carbon cloth and a piece of insulator (plastic)-coated copper wire, both of which are supported by a three-dimensional rack made of wood sticks. The two electrodes are connected via a dc voltage source V_appl_ (a series assembly of 1.5 V batteries connected to a variable resistor) and an ammeter. This arrangement precludes that V_appl_ causes current in the circuit. The temperature of the system is maintained by irradiating light on the glass jar using an electric lamp and the temperature of the fermentation mixture is monitored using a thermometer dipped into the fermentation mixture. The fermentation mixture is prepared by mixing dry yeast (*S. cerevisiae*), glucose with de-ionized water. The mixture is used immediately. Dry nitrogen (99.999%) is used to purge oxygen in the mixture for 30 minutes. Then the glass container is sealed for fermentation. Magnetic stirring is used ensure the homogeneity of the mixture. A syringe is used to transfer samples/reagents during the fermentation. The pH of the mixture is maintained at 5.

### Reagents and materials

Dry *S. cerevisiae* (baker’s yeast) was purchased from Sigma Aldrich (YSC1-100G) and a grocery store (Baker’s Corner or Fleischmann’s). Both yeasts gave similar results. Glucose was purchased from Sigma Aldrich (G7528-1 KG). De-ionized water (18.2 MΩ-cm) was used in the present work. Peptone was purchased from Fluka Analytical (70173-100 G). Carbon cloth was made by Fuel Cell Earth (Product code: CC20WP05).

### Spectrophotometer and assay kits

A spectrophotometer made by Thermo Scientific (Genesys 10S UV-VIS) was used to determine the concentration of glucose and ethanol. Assay kits for glucose and ethanol were purchased form Sigma Aldrich (Glucose(HK) Assay Kit, GAHK20-KT) and BioAssay Systems, USA (Quantichrom Ethanol Assay, DIET-500), respectively. The spectrophotometer was also used to determine the optical density of the growth of *S. cerevisiae.*

### Glucose measurement

BREEZE®2 blood glucose test strips and a BREEZE®2 blood glucose metre (Bayer Health Care, Mishawaka, WI) with a measuring range of 20–600 mg/dL (1.11–33.33 mM) were also used to measure the concentration of glucose in samples. The metre was calibrated before use.

### Ethanol measurement

The ethanol concentration of samples (%v/v) was also measured using an ebulliometer (Dujardin-Salleron, Paris, France) at room temperature.

## Additional Information

**How to cite this article**: Sam Mathew, A. *et al.* Enhanced ethanol production via electrostatically accelerated fermentation of glucose using *Saccharomyces cerevisiae*. *Sci. Rep.* **5**, 15713; doi: 10.1038/srep15713 (2015).
